# From tumor microenvironment to emerging biomarkers: the reshaping of the esophageal squamous cell carcinoma tumor microenvironment by neoadjuvant chemotherapy combined with immunotherapy

**DOI:** 10.3389/fimmu.2024.1478922

**Published:** 2024-12-05

**Authors:** Zhengzhou Qiu, Zhao Li, Xingfei Liu, Ruilin Zhang, Yongxuan Li, Chenggen Gao, Xiaoling Mao, Yin Bao, Mingyue Zhang, Changying Guo

**Affiliations:** ^1^ Jiangxi Medical College, Nanchang University, NanChang, China; ^2^ Department of Thoracic Surgery, Jiangxi Cancer Hospital, The Second Affiliated Hospital of Nanchang Medical College, Jiangxi Cancer Institute, Nanchang, China; ^3^ Jiangxi Key Laboratory of Oncology, Jiangxi Cancer Hospital, Nanchang, Jiangxi, China; ^4^ Medical College, Jiangxi University of Chinese Medicine, Nanchang, Jiangxi, China; ^5^ Zhejiang-Jiangxi Joint Thoracic Oncology Research Laboratory, Jiangxi Cancer Hospital, Nanchang, Jiangxi, China

**Keywords:** esophageal cancer, esophageal squamous cell carcinoma (ESCC), immunotherapy, neoadjuvant therapy, tumor microenvironment (TME), tumor immune microenvironment, tumor-infiltrating lymphocytes (TILs), biomarkers

## Abstract

Esophageal squamous cell carcinoma is a cancer with high morbidity and mortality. The advent of immune checkpoint inhibitors has significantly increased complete response rates and postoperative R0 resection rates after neoadjuvant therapy. These drugs can largely reverse the suppression of the immune system caused by the tumor microenvironment, allowing the reactivation of anti-tumor immune infiltrating cells, significantly improving the patient’s tumor microenvironment, and thus preventing tumor development. However, there are still some patients who respond poorly to neoadjuvant combined immunotherapy and cannot achieve the expected results. It is now found that exploring changes in the tumor microenvironment not only elucidates patient responsiveness to immunotherapy and identifies more reliable biomarkers, but also addresses the limitations of prediction with imaging examination such as CT and the instability of existing biomarkers. In light of these considerations, this review aims to delve into the alterations within the tumor microenvironment and identify potential predictive biomarkers ensuing from neoadjuvant immunotherapy in the context of esophageal squamous cell carcinoma.

## 1 Introduction

Esophageal cancer has a high incidence and mortality rate, with esophageal squamous cell carcinoma(ESCC) being the predominant histological subtype (1–3). Neoadjuvant chemotherapy is widely applied in the perioperative management of ESCC, yet the outcomes have not been entirely satisfactory. Since the introduction of immune checkpoint inhibitors (ICI), they have been extensively applied in the treatment of various cancers. In the field of ESCC, numerous large-scale clinical trials have shown that neoadjuvant immunotherapy improves the pathological response rate(pCR) and extends patient survival. Moreover, neoadjuvant chemotherapy and immunotherapy (nCIT) can increase the chances of surgery and enhance the rate of R0 resections, offering hope to patients.

**Figure 1:**
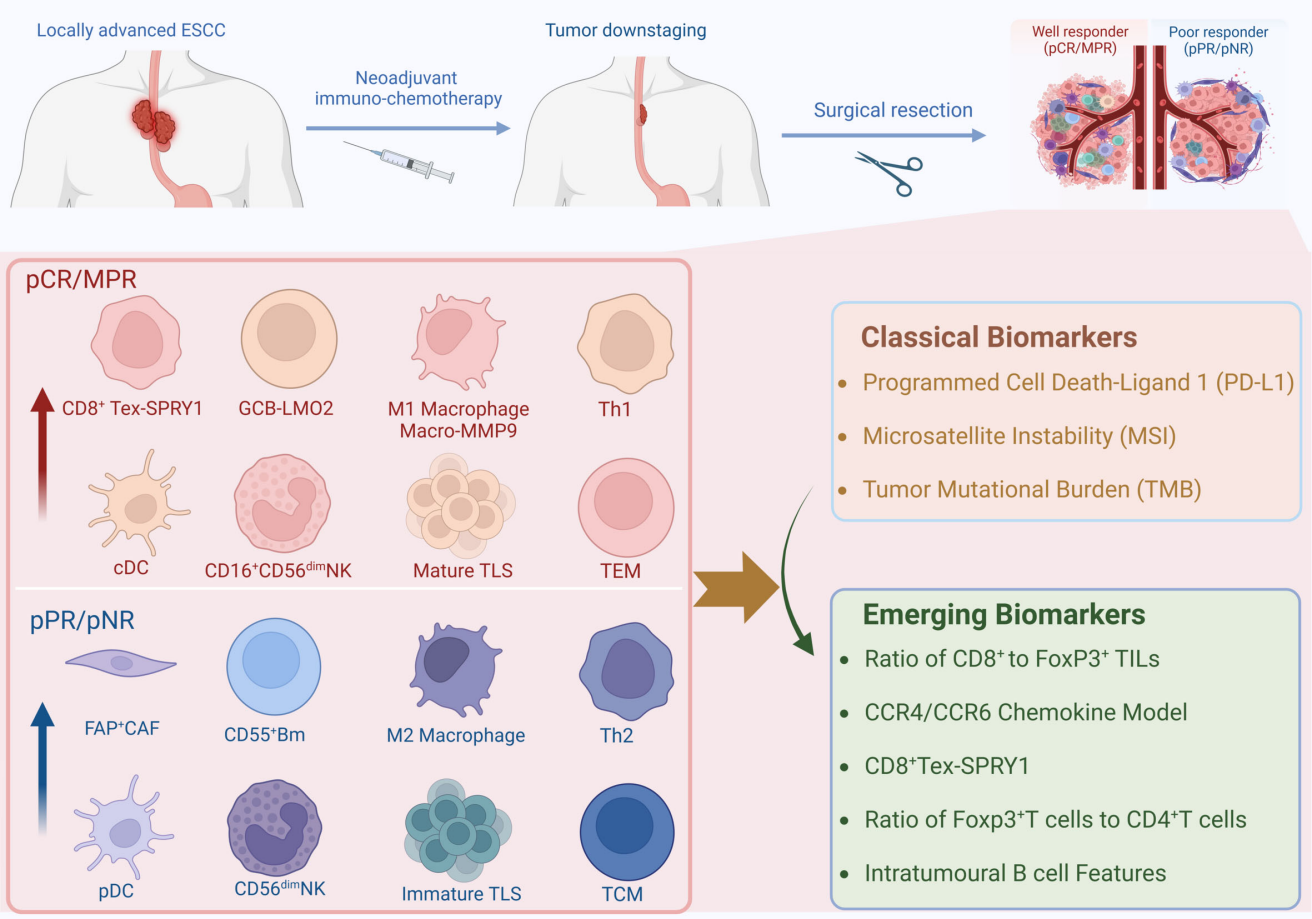
Graphical Introduction. Created in BioRender. Ka, X. (2024) https://BioRender.com/v51n660.

Surgery following neoadjuvant chemoimmunotherapy (nCIT) is considered the optimal treatment approach (4). However, because esophageal cancer is a hollow viscus tumor, there are certain limitations when using the immune-related Response Evaluation Criteria in Solid Tumors (iRECIST) to assess the efficacy of neoadjuvant therapy before surgery for resectable esophageal cancer. The invasiveness of endoscopy and its costs also restrict the widespread application of endoscopic assessment (5). Concurrently, there remains a subset of patients who do not benefit from this combined therapy, delaying surgical opportunities and leading to a poorer prognosis (6, 7). Therefore, it is significant to investigate the alterations and potential biomarkers in the tumor microenvironment (TME) of ESCC following nCIT. Recently, Existing biomarkers predictive of nCIT efficacy, including the tumor proportion score (TPS) for PD-L1, have shown limited sensitivity or specificity in predicting therapeutic outcomes (8). Researchers are increasingly focusing on the impact of TME after nCIT and utilizing Single-cell RNA-sequence to analyze various immune cells. Thus, cells and genes with significant alterations are expected to become more specific and sensitive biomarkers. Therefore, We provide a brief overview of the current status of research on nCIT, summarize the alterations and potential biomarkers on the TME of ESCC after nCIT, and discuss future research directions in this field (**Figure 1**).

## 2 Current status of neoadjuvant immunotherapy research

Following the introduction of neoadjuvant therapy, large-scale clinical trials, such as the CROSS trial (9) and the NEOCRTEC5010 trial (10), have established neoadjuvant chemoradiotherapy (nCRT) as a first-line treatment new option for resectable locally advanced esophageal cancer. Furthermore, clinical studies including CheckMate 648, ESCORT, and KEYNOTE-181 have revealed the excellent efficacy of immune checkpoint inhibitors (ICIs) in unresectable advanced or recurrent esophageal cancer, prompting researchers to focus on the nCIT (11–13).

At present, prospective studies, such as NICE (14)and Neo-PLANET (15), have demonstrated excellent pathological complete response (pCR) rates for nCIT in esophageal squamous cell carcinoma (ESCC), ranging from 21.2% to 40.0% (16). Xu et al. included 9 trials in a meta-analysis, pointing out that the pCR rate of nCIT significantly reached 26.9% (OR, 4.24; 95% CI, 2.84-6.32), which is more than three times higher than the 8.3% observed in the group receiving chemotherapy alone (nCT) (17). Similarly, in the field of lung cancer, Sorin et al. included 43 trials in a meta-analysis and indicated that in comparison with nCT, nCIT has significantly increased the pCR rate to the range of 20% to 40%(RR, 5.52; 95% CI, 4.25-7.15) (18). In the gastric cancer field, the combined pCR rate increased to 24% (95% CI: 19%–28%) (19). In the field of melanoma, the pCR rate increased to 57%, with a 2-year RFS range of 94% to 100% (20). Regarding the pCR rate of ESCC is lower than that of melanoma, most researchers believe that melanoma has a higher immunogenicity, making immune cells easily activated and having a good response rate. However, the efficacy of ESCC is similar to that of NSCLC and gastric cancer, with significant improvements including other observed indicators such as pCR, MPR, and OS. At the same time, these studies indicate that nCIT has controllable treatment-related toxic effects, and the rate of R0 resection after surgery has also increased, further demonstrating that nCIT is a better choice for neoadjuvant therapy. To this end, we have also summarized the ongoing clinical trials on neoadjuvant therapy for ESCC, as shown in **Table 1**.

**Table 1:** Ongoing clinical trials of neoadjuvant immunotherapy for resectable ESCC.

Trial No.	Phase	Country	Design	Targeted ICIs	Start date
**FRONTiER** NCT03914443	I	Japan	Two-arm	Nivolumab	2019-04-11
**NICE-2** NCT05043688	II	China	Three-arm	Camrelizumab	2021-09-01
**NICCE** NCT05028231	NA	China	Single-arm	Sintilimab	2021-06-05
**NEOCRTEC2101** NCT05357846	III	China	Two-arm	Sintilimab	2022-11-01
**JS001** NCT04848753	III	China	Two-arm	Toripalimab	2021-06-23
**iCROSS** NCT04973306	II-III	China	Two-arm	Tislelizumab	2022-03-02
**NICE-RT** NCT05650216	II	China	Single-arm	Camrelizumab	2022-12-25
**ETNT** NCT05189730	II	China	Single-arm	Tislelizumab	2021-07-01
**NATION1907II** NCT04215471	II	China	Single-arm	SHR-1316	2022-07-01
**KEYSTONE-002** NCT04807673	III	China	Two-arm	Pembrolizumab	2021-12-01
**REVO** NCT05007145	II	China	Two-arm	PD-1 Inhibitor	2021-08-15
NCT05281003	II	China	Single-arm	Pembrolizumab	2023-02-20
NCT04520035	II	China	Single-arm	Camrelizumab	2020-08-20
NCT04767295	II	China	Single-arm	Camrelizumab	2021-03-01
NCT05213312	II-III	China	Two-arm	Nivolumab	2022-06-01
NCT05476380	II	China	Single-arm	Camrelizumab	2021-02-19
NCT05182944	II	China	Four-arm	Camrelizumab	2022-01-15
NCT04937673	II	China	Two-arm	Camrelizumab	2021-07-01
NCT05176002	I-II	China	Single-arm	Camrelizumab	2021-09-23
NCT04666090	II	China	Single-arm	Camrelizumab	2020-11-23
NCT05355168	I-II	China	Single-arm	Camrelizumab	2021-11-01
NCT05244798	III	China	Three-arm	Sintilimab	2022-11-01
NCT04280822	III	China	Two-arm	Toripalimab	2020-04-21
NCT04804696	II	China	Single-arm	Toripalimab	2021-02-10
NCT04888403	II	China	Single-arm	Toripalimab	2021-12-31
NCT04644250	II	China	Single-arm	Toripalimab	2020-09-01
NCT05323890	II	China	Single-arm	Tislelizumab	2022-04-20
NCT04974047	II	China	Two-arm	Tislelizumab	2021-08-17
NCT 04437212	II	China	Single-arm	Toripalimab	2020-07-01
NCT05424432	II	China	Single-arm	Toripalimab	2022-04-29
NCT06225921	I	China	Two-arm	Adebrelimab& Dalpiciclib	2023-12-29
NCT06637163	II	China	Two-arm	Benmelstobart	2024-10-01
NCT05659251	II	China	Single-arm	Serplulimab	2024-10-01
NCT06508229	II	China	Single-arm	Adebrelimab	2024-07-30

NA, Not available. The bolded term, such as "FRONTiER," refers to the acronym of the title provided at the time of registration for this large-scale clinical study.

## 3 Alterations in the tumor microenvironment

Numerous studies indicate a potential correlation between TME and pathological response (21, 22). In the TME, immune checkpoint blockade (ICB) or immune checkpoint inhibitor (ICI) targets immunosuppressive molecules of tumor cells, thereby reactivating exhausted T cells to resist tumor cells (23). However, the composition of the TME in ESCC is complex. Although tumor-infiltrating CD8^+^ T cells and NK cells exert antitumor effects, the regulatory T cells (24) and M2 macrophages exert immunosuppressive effects, leading to deficiencies in immune surveillance (25). Furthermore, extensive fibrosis and extracellular matrix deposition also augment the tumor’s resistance to immunotherapy (26). Post-treatment with ICB, the interactions and changes among various cells within TME remain insufficiently elucidated. The response to immunotherapy does not always correlate with PD-L1 expression (27, 28). Therefore, it is crucial to explore the alteration of TME before and after immunotherapy and identify relevant biomarkers.

### 3.1 CD8^+^ T cell

CD8^+^ T cells are a crucial subset of T cells, showing the most significant difference after neoadjuvant chemoimmunotherapy (nCIT) (29). CD8^+^ T cells are T cells that have undergone differentiation and maturation to express CD8 molecules. They can secrete large amounts of IFN-γ and granzyme B to synergize against cancer cells (30). Numerous studies have found that nCIT increases CD8^+^ T cell infiltration more significantly than neoadjuvant chemotherapy (28), prolonging overall patient survival (31). And the same trend can be observed before and after nCIT, whereas regulatory T cells (Treg) show the opposite trend. One of the studies has indicated that the density of CD8^+^ tumor-infiltrating lymphocytes (TILs) in the pCR and major pathological response (MPR) groups is higher than in the pathological partial response (pPR) and pathological non-response (pNR) groups. Additionally, the ratio of CD8^+^ to FoxP3^+^ TILs in the pCR/MPR group is significantly greater than in the pPR/pNR group, suggesting that this ratio could emerge as a novel biomarker after nCIT in ESCC. Interestingly, in the patients not achieving pCR, CD8^+^ TILs were observed to be mostly distributed in the periphery of the tumor, correlating with a worse prognosis, while the same cellular distribution was found in the lymph nodes (32).

Based on the expression of antigenic clusters, CD8^+^ T cells are classified into different T cell subpopulations. In addition to the regulatory CD8^+^ T cell subset (33), there are Tc1 cells with the most typical cytotoxic function (34). In the tumor microenvironment, CD8^+^ T cells generally resist cancer cells by differentiating into cytotoxic T cells (29). However, T cells chronically exposed to persistent antigens or chronic inflammatory environments are prevented from acquiring memory cell homeostatic responsiveness (34–36). The immediate protection and recall response to antigens conferred by memory lymphocytes constitutes a critical component of the organism’s immune defense (37). Nowadays, studies have shown a decrease in the ratio of central memory cells (TCM) to effector memory T cells (TEM) in samples of patients who have achieved pCR/MPR, reversing the high pre-treatment TCM/TEM ratio (28). The process of TCM-to-TEM, which occurs on antigenic restimulation, produces greater anti-tumor capacity (38, 39).

**Figure 2:**
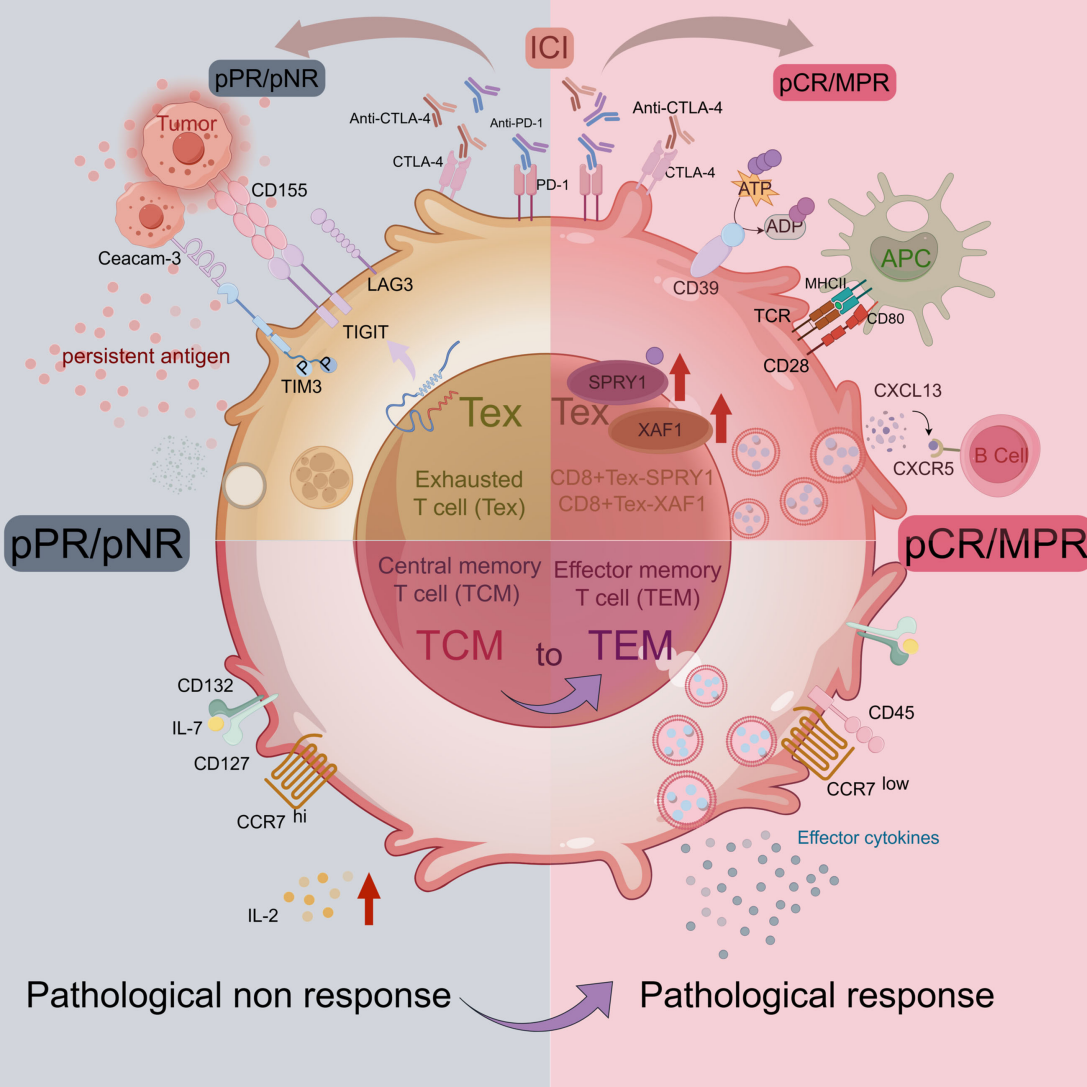
T cell in TME of ESCC. Image created with figdraw.com,ID: IUIUT533a6.

T cells, during their process of progressive exhaustion, upregulate the expression of inhibitory pathway molecules, including PD-1 and CTLA-4 (40). Thus, ICB facilitates the reactivation of exhausted CD8^+^ T cells by blocking the inhibitory molecules. However, with an in-depth study, it was found that even with ICB, a large number of exhausted T cells (Tex) still existed in the TME (41) and it could be divided into ICI-permissive and ICI-resistant subpopulations (42, 43). However, two clusters of cells, CD8^+^ Tex-SPRY1 and CD8^+^ Tex-XAF1, were found to respond significantly to ICI treatment. Furthermore, the anti-tumor signature genes ENTPD1, CXCL13, and HLA-DR genes were enriched in these two CD8^+^ Tex subsets. The CD8^+^ Tex-SPRY1 cells were in the early stage of exhausted T cells and showed high similarity to progenitor CD8^+^ T cells. Moreover, these samples from patients who achieved pCR had higher expression of SPRY1 and CD8 in the tumor stroma and intra-tumoral regions before and after nCIT, which was not observed in patients who did not achieve pCR (**Figure 2**). It is suggested that patients with high CD8^+^ Tex-SPRY1 cell infiltration have a higher sensitivity to nCIT and that CD8^+^ Tex-SPRY1 may be a potential biomarker for nCIT in ESCC.

### 3.2 CD4^+^ T cell

CD8^+^ T cells showed a significant correlation with nCIT, but the heterogeneity of the TME also limits its stability as a biomarker. CD4^+^ T cells, unlike CD8^+^ T cells that can directly kill tumor cells, function mainly by releasing different cytokines to regulate other immune cells and play an equally important role as CD8^+^ T cells. CD4^+^ T cells represent a heterogeneous subset of T cells, and different levels of CD4^+^ T cell numbers are associated with different outcomes. Some studies have noted that a significant increase in CD4^+^ T cells is observed after nCIT compared to neoadjuvant chemotherapy alone and that higher CD4^+^/CD8^+^ ratios are associated with better pathological response (44). However, it has also been noted that the high level of CD4^+^ T cell distribution within a distance of 0-10 μm from the tumor cells predicts a low survival rate (45). Tumor-infiltrating CD4^+^ T cells exert their function by undergoing differentiation into various subsets directed by functional polarization. Moreover, the differentiation of these subsets is significantly influenced by the characteristics of the TME in which they exist. For example, Th1 cells exert antitumor effects, whereas Th2 cells counteract the effects of Th1 cells, and the Th9 is unchanged after nCIT (28).

Recently, a single-cell sequencing study classified CD4^+^ T cells into six major subsets based on specific markers. Among them, CD4-C1-CCR7 carries naïve signature genes, including TCF7, CCR7, LEF1, and SELL. CD4-C6-FOXP3 has the most distinctive Treg profile, expressing high levels of the Treg signature genes FOXP3, IKZF2, IL2RA, and CTLA-4. The study noted that Tregs are heavily enriched in ESCC and that Tregs accounted for more than 50% of the total CD4^+^ T cells in the tumors, compared to only 25% in the adjacent normal tissues (46). At the same time, numerous studies have indicated that Treg cells can significantly inhibit T cell-mediated antitumor effects (47). The density of Treg cells in ESCC tissues was higher than that in normal tissues. However, after nCIT, the number of Treg decreased while CD8^+^ T cells increased (28). FoxP3^+^ TILs were found in higher quantities in tumor samples from patients who achieved pNR compared to those who achieved pCR (32). It was also found that there exists a subset of T cells expressing CD4^+^CD25^-^CD69^+^Foxp3^-^LAP^+^, which inhibits T cell proliferation and promotes tumor immune escape by secreting TGF-β. This subset was positively correlated with the pathologic response (48). However, only high Treg density in ESCC tissues is not predictive of patient survival (45). The calculation of the ratio between Treg and CD4^+^ T cells is required to observe a positive correlation with better pathological response (49). Thus, the ratio of FoxP3^+^ T cells to CD4^+^ T cells is expected to be a biomarker for predicting the response to nCIT, as mentioned previously for CD8^+^ TILs/FoxP3^+^ TILs.

### 3.3 Macrophage

Macrophages are known as the “scavengers” of the human organism. They can not only phagocytose pathogens but also exert the function of presenting antigens by processing antigens to form MHC complexes. Macrophages migrate into the TME and become tumor-associated macrophages (TAMs), which play an important role in the initiation and regulation of antitumor immune responses (50). Recently, it was found that dendritic cells and macrophages were mostly distributed within 30 μm from tumor cells and that patients with closer spatial distance of TAM’s distribution tend to have better immune responses and longer survival (45). This is because chemotherapy causes tumor cells to release more tumor-associated antigens, and the closer the distance between TAMs and tumor cells, the better for TAMs to be exposed to these more new antigens, which provide immunomodulatory signals to the T cells to promote anti-tumor immune responses (51). This suggests that spatial distances between TAMs and tumor cells are potential predictive biomarkers (52).

TAMs can be activated by M1 and M2 types. M1-TAMs exert proinflammatory and antitumor effects (53), enhancing the antigen-presenting capacity of DC and augmenting tumoricidal effects of infiltrating T cells (54). Tumor tissues in the pCR group were observed to have a significant M1 polarization level before treatment and a significantly higher M1/M2 ratio than in the non-pCR group after treatment (29, 55). Among M1-type macrophages, Macro-MMP9 and Macro-FOLR2 subset produced large amounts of cytokines such as IL-15, CXCL1, and CXCL9, which induced the activation of CD8^+^ Tex-SPRY1 subset. Thus Macro-MMP9 and CD8^+^ Tex-SPRY1 form a positive feedforward cycle and anti-tumor ability (29). Whereas M2-TAMs could inhibit M1-TAMs effects to promote immunesuppression (56), the number of PD-L1^+^CD163^+^ cells infiltrated was significantly increased in the non-pCR group (57). Recently, it has been shown that macrophage migration inhibitory factor (MIF) promotes the conversion of M1-type TAMs to M2-type, and patients with high baseline MIF levels are significantly associated with adverse pathologic responses.

**Figure 3:**
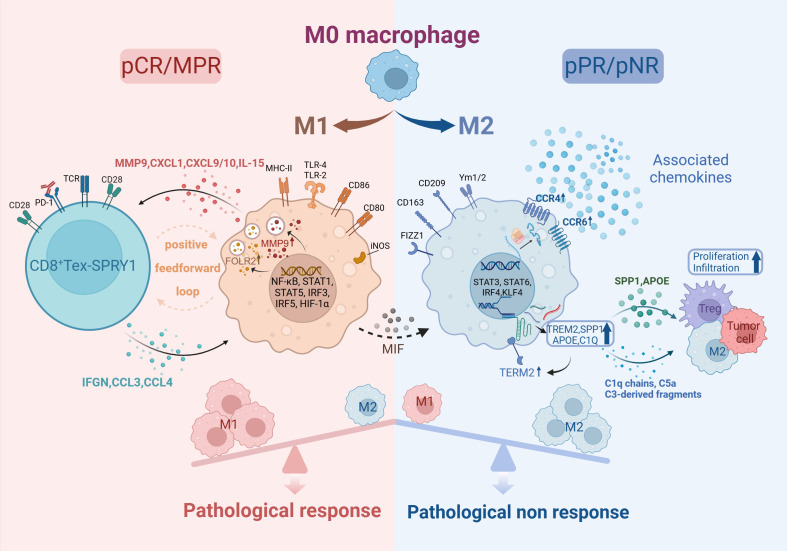
Macrophage in TME of ESCC. Created in BioRender. Q, Z. (2024) https://BioRender.com/b87g297.

Macrophages exhibit high plasticity and heterogeneity (58). CCR4^+^CCR6^+^ TAM were found to be significantly reduced after nCIT, while the percentage of total macrophages in the tumor microenvironment was not changed. The CCR4/CCR6 chemokine system score was lower in tumor regression grading (TRG) of grade 0/1 compared to patients with TRG2/3, suggesting that the CCR4/CCR6 chemokine system would be a potential biomarker (28). Another subset of TAM, TAM-TREM2, has an immunosuppressive phenotype and is functionally close to the M2 subtype (59). Their high level of infiltration is associated with worse OS (60). Further studies showed that TAM-TREM2 expressing key genes of the complement system, C1Q, APOE, and SPP1, promote infiltration of Tex and Tregs as well as tumor proliferation (61) (**Figure 3**). Meanwhile, the TIDE algorithm predicted that signature genes of TAM-TREM2 are associated with immune resistance. It is proposed that this subset is expected to be a predictive biomarker for nCIT (60).

### 3.4 Dendritic cell

In TME, dendritic cells (DCs), similar to macrophages, exert antigen-presenting function and regulate the immune response, thereby influencing tumor immune surveillance and escape (62). Among long non-coding RNAs, lncRNA ENST00000560647 has been shown to inhibit dendritic cell-mediated antigen presentation, leading to the inactivation of CD8^+^ T cells and the facilitation of immune evasion (63). DCs can differentiate into classic dendritic cell cDC and plasma cell-like dendritic cells pDC in TME (64). The current study found that the density of pDC in tumor tissues of patients not treated with nCIT is higher than in adjacent normal tissues, and then significantly decreased after receiving nCIT. This is consistent with the fact that pDCs produce IFN-α which leads to differentiation of Tregs and immune escape (65). The TCGA database survival analysis also illustrated that the characterized genes of pDC can differentiate patient survival well. Another DC subset, cDC, whose quantitative changes are mainly influenced by classical cDC type 1. cDC1 can activate CD4^+^ and CD8^+^ effector T cells, which are potentially responsive to immunotherapy (66). Therefore, the regulation of DC to exert its antitumor effects has become one of the hotspots in immunotherapy, including the use of tumor vaccines. Tumor vaccines utilize anti-CLEC9A antibodies to specifically deliver the immunogenic tumor antigen NY-ESO-1 to human CD141^+^ DC cells for anticancer therapy (67). Meanwhile, overexpression of MAGE-A3 and CALR on DCs to study their potential for generating anti-tumor immune responses is also one of them (68).

### 3.5 Natural killer cell

NK cells are the core cells of the organism’s innate immune cells. NK cells have a broader-spectrum immune role that not only is limited to fending off viruses but also has a crucial role in killing tumor cells (69). CD56^+^ is the characteristic gene of NK cells, and NK cells can be further categorized into two major subsets based on CD56 expression: CD56^bright^ and CD56^dim^ NK cells (70). CD56^bright^ NK is the precursor cell of CD56^dim^ NK (71), but CD56^dim^ NK expresses higher levels of FCγ receptor III (CD16) and possesses stronger natural cytotoxicity and antitumor effects (72, 73). The present study found that CD16^+^ cells were less in tumor tissues than in normal tissues and were significantly higher after nCIT, while CD16^-^ cells exhibited higher exhaustion scores (28). Meanwhile, CD56^dim^ NK cells were significantly reduced in the responders compared to non-responders within the nCIT group, whereas no significant change was observed in the neoadjuvant chemotherapy group. And, the pCR group had more abundant CD56^dim^ NK cells in the stromal area than the non-pCR group. These results show that CD16^+^CD56^dim^ NK cells are associated with a better pathological response (55). ICB could activate numerous PD-1-expressing NK cells (74, 75), and CD16 could strongly self-activate NK cells (76). These all support an immunotherapeutic approach using modified NK cells to resist tumors, known as chimeric antigen receptor-NK (CAR-NK) cell therapy.

CAR-NK cell therapy, from adoptive cell therapy (ACTs), involves the transduction of a specially designed chimeric antigen receptor into NK cells. This enables CAR-NK cells to specifically recognize antigens on the surface of tumor cells and be activated, subsequently releasing cytotoxic molecules to eliminate tumor cells (77). Research on adoptive cell therapy, which began with tumor-infiltrating T cells, has demonstrated promising efficacy in hematological malignancies. However, the development of CAR-T therapy is constrained by issues related to infiltration into tumor tissues, as well as resistance to the tumor microenvironment (TME). Compared to CAR-T therapy, CAR-NK therapy can reduce the cytokine release syndrome (CRS) and neurological toxicities associated with CAR-T treatment (78). These advantages position CAR-NK as a potential “universal” product (79). CD19, CD20, and CD33 are the main targets for CAR-NK cells, which are frequently expressed in hematological malignancies (80). Additionally, CAR-NK cells have been shown to be effective against solid tumor targets such as Her2, EpCAM, and EGFR, which are common in colorectal and ovarian cancers (81–83). This broadens the applicability of CAR-NK cell therapy. Currently, CAR-NK cell therapy faces challenges such as viral transduction difficulties. Researchers are developing CAR-NK cells capable of recognizing multiple antigens to reduce the risk of tumor escape due to the loss of a single antigen (84). Moreover, CAR-NK cell therapy is being explored in combination with other treatment modalities to expand the applicability of immunotherapy. It is anticipated that ACTs will also become a milestone in immunological treatment.

### 3.6 B cells and tertiary lymphoid structure

**Figure 4:**
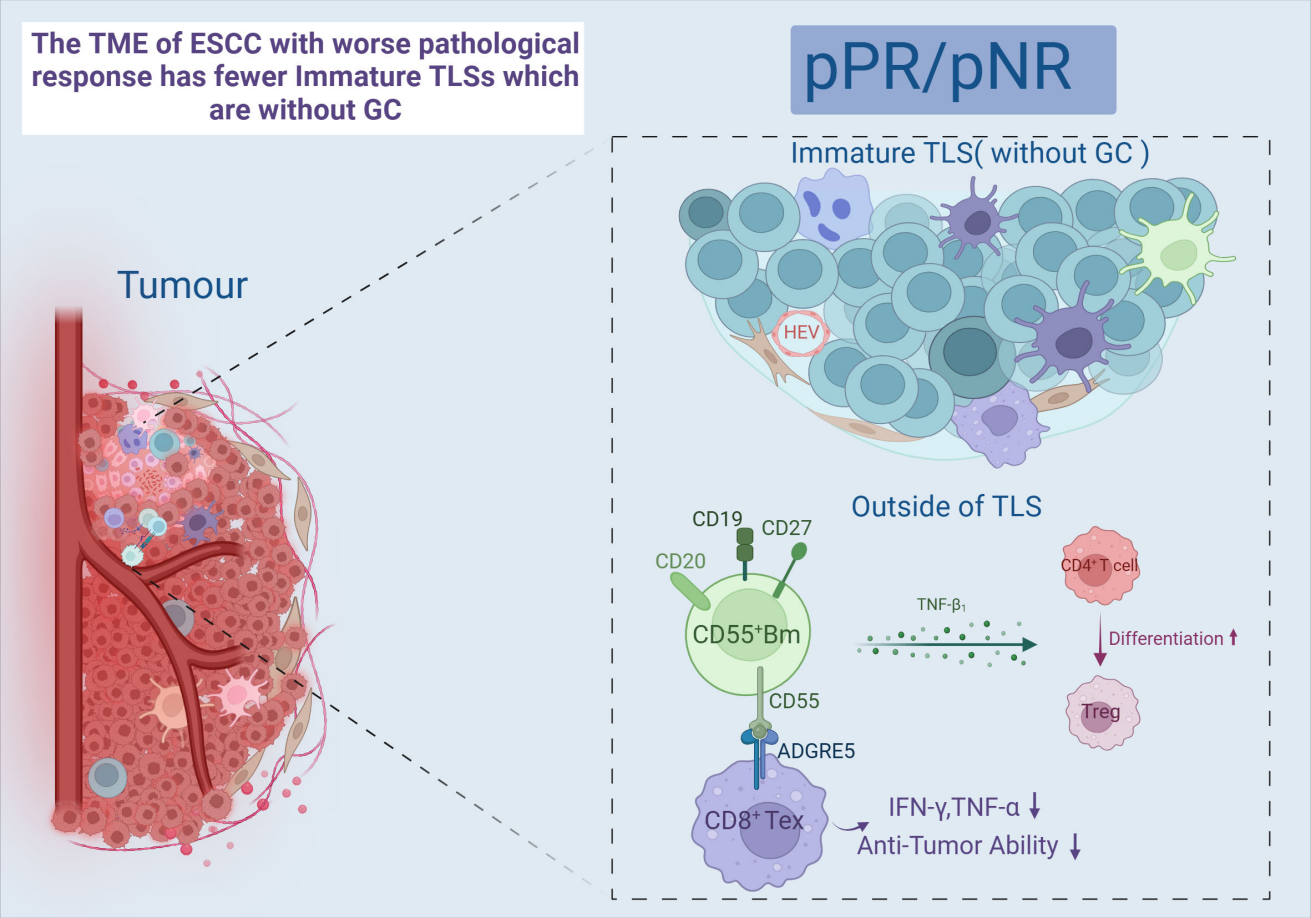
Immature TLS and CD55^+^Bm. Created in BioRender. Q, Z. (2024) https://BioRender.com/a41h264.

B cells are the major humoral immune cells, exerting antibody-dependent cytotoxicity (ADCC) and antibody-dependent cellular phagocytosis (ADCP) (85). There is growing evidence that tumor-infiltrating B cells (TIL-B) and plasma cells have a crucial role in immunomodulation (86). One study showed a significant infiltration of B cells in a subgroup of “immune activation-dominant” samples. COL19A1, as a receptor for TIL-B, positively correlates with pathological responses and is expected to be a biomarker predicting the prognosis of nCIT (87). The present study classified TIL-B into five major subsets: naïve B cells (NBCs); activated B cells (ABCs); memory B cells (MBCs); germinal center B cells (GCBs), and the ASC (88). Two of these B cell subsets are sensitive to immunotherapy, GCB-LMO2 and CD55^+^ Bm. First, patients with a pathological response showed higher GCB cell infiltration than pathological non-response patients, and a significant increase in the interaction between the GCB-LMO2 and the CD4^+^ T-CXCL13 subsets was observed. GCB-LMO2 expresses the conventional GCB cell markers GL-7 and CD23, which are distributed within TLS and promote an antitumor immune response. CD55^+^ Bm belongs to the group of memory B cells that express relevant genes such as CD20, which are distributed outside the TLS. The expression of its signature gene, CD55, is down regulated with increased expression of LMO2. CD55^+^ Bm expresses ADGRE5, which inhibits CD8^+^ T cells and releases cytokines that interact with Treg cells to inhibit responsiveness to immunotherapy (**Figure 4**). It is illustrated that comprehensive B cell profile characteristics can predict nCIT responsiveness (89).

**Figure 5:**
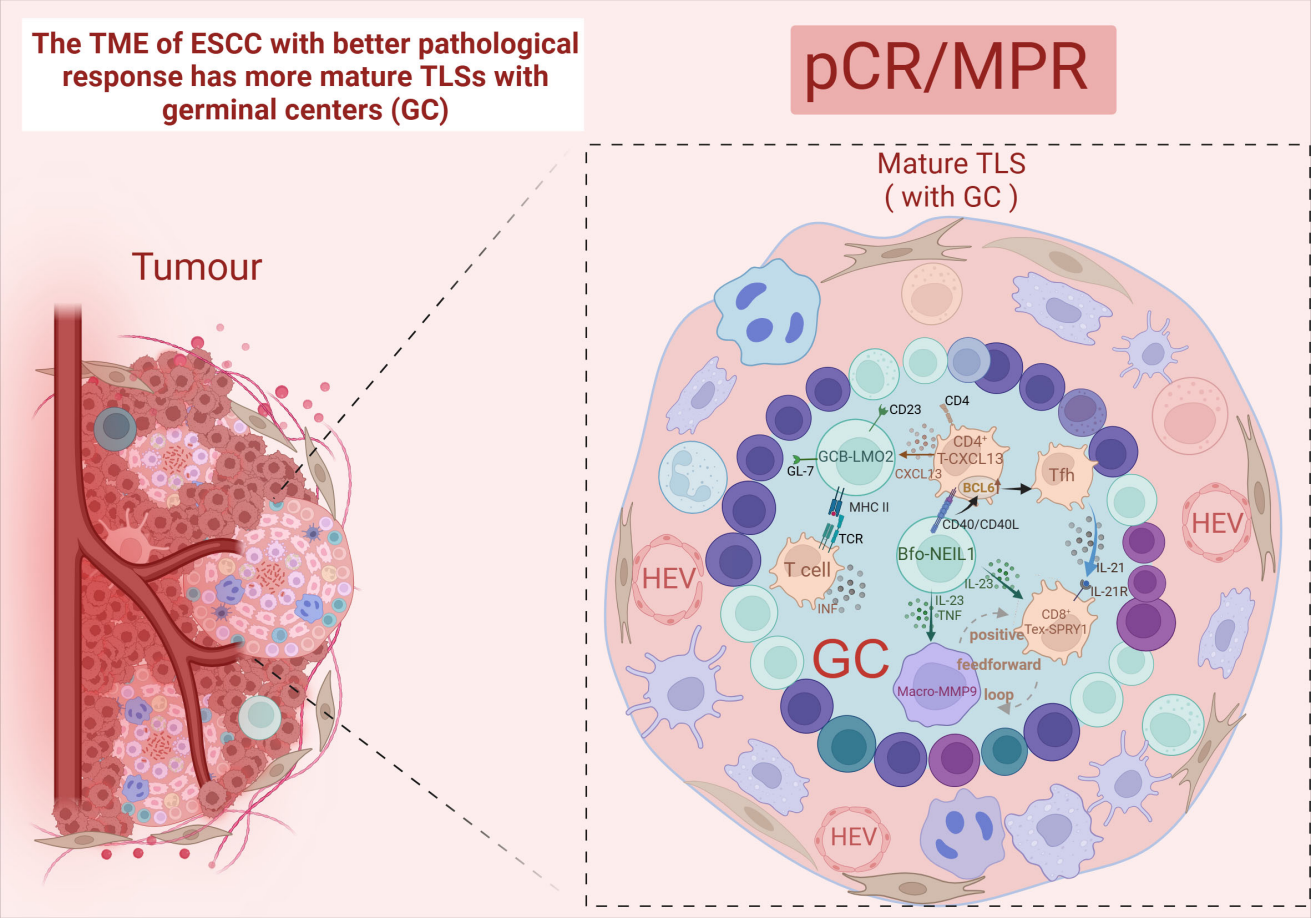
Mature TLS. Created in BioRender. Q, Z. (2024) https://BioRender.com/l91q142.

Tertiary lymphoid structure (TLS) refers to the organization of immune cell aggregates that resemble lymphoid tissues, formed within non-lymphoid tissues. In the physiological state, TLS is not present in normal tissues and is mainly found in the infiltrative margins of tumors (90). In recent years, TLS has been found to have a positive effect on the prognosis of many cancers, including lung cancer (91) and ovarian cancer (92). However, both RFS and OS were higher in the TLS-positive group than in the TLS-negative group, and the mature TLS group containing germinal centers (GCs) had better long-term efficacy and prognosis (93, 94). After nCIT, TME with abundant TLS was the most common type among the patients who achieved pCR or MPR (95, 96). Moreover, a study has developed a pathology image model using deep learning to quantitatively calculate the TLS ratio for predicting the pathological response to nCIT in ESCC. In TME, TLS is constructed by GCBs, T cells, and B cells (7). And it can only be activated in the presence of GCB-LMO2 cells. Meanwhile, patients with TLS high in CD55^+^ Bm cells and low in GCB-LMO2 cells were stratified as having the worst clinical outcome (89). This indicates that the antigen-presenting role of GCB cells contributes to the maturation of cytotoxic T cell (97). Follicular B cells (Bfo-NEIL1), which originate from GCB and express NEIL1, co-localize with CD8^+^ Tex-SPRY1 within TLS and exhibit better pathological responses. This co-localization demonstrates a synergistic interaction that significantly improves RFS. Bfo-NEIL1 cells express TNF and IL-23 to activate CD8^+^ Tex-SPRY1 and promote the positive feedforward cycle between Macro-MMP9 cells and CD8^+^ Tex-SPRY1. Additionally, CD4^+^ T-CXCL13 cells were induced by B-T interaction to activate the Tfh phenotype, which in turn activated CD8^+^ Tex-SPRY1 again. (**Figure 5**) Meanwhile, CD8^+^ Tex-SPRY1 induced the activity of germinal centers and the TLS, coinciding with the recent studies that the CD8^+^ response is present in the TLS (90, 98).

## 4 Interactions and spatial distribution of immune cells

**Figure 6:**
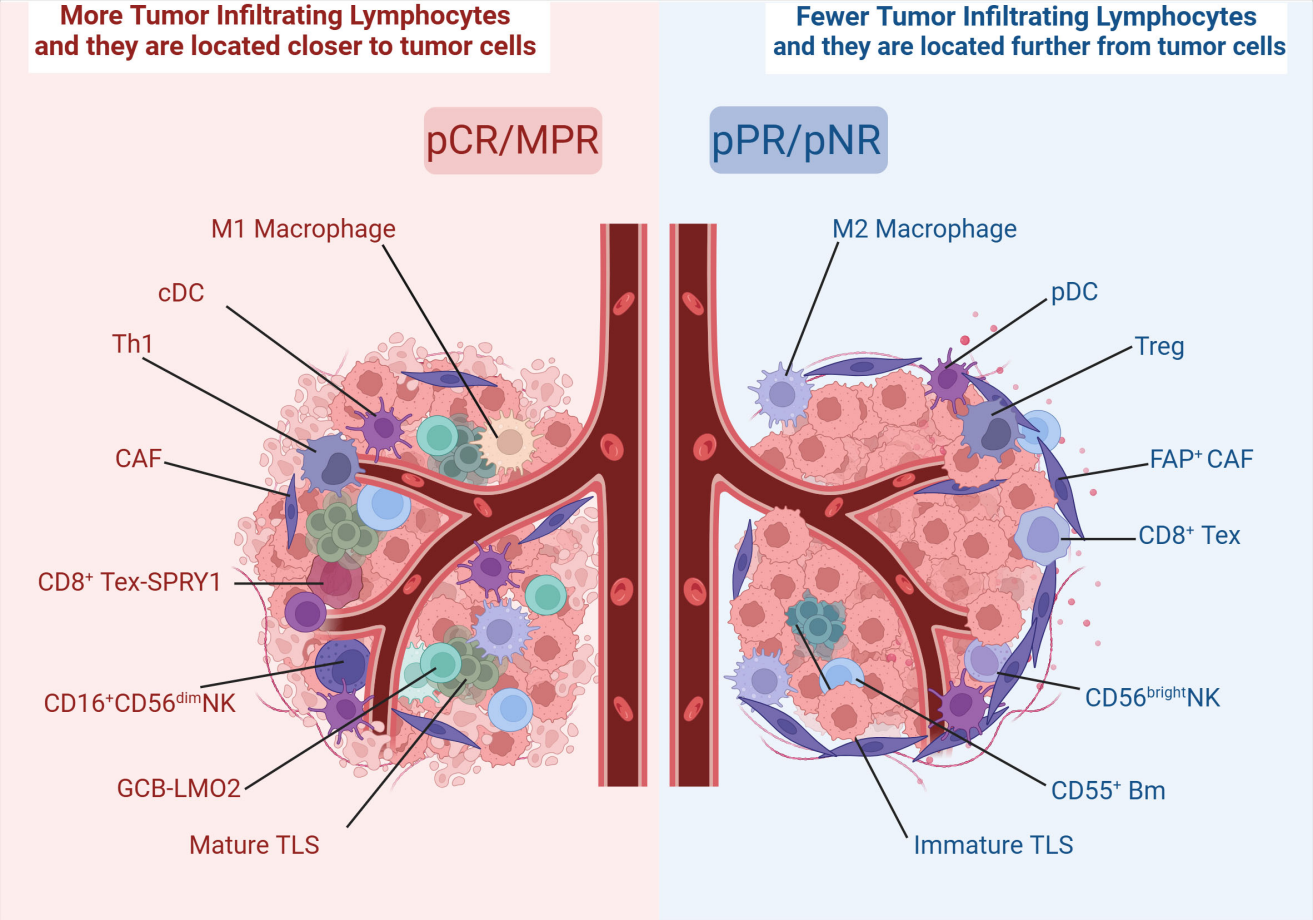
Different distribution of immune cells in TME. Created in BioRender. Q, Z. (2024) https://BioRender.com/r37r267.

It is well known that tumors can be viewed as complex systems (99). The response of tumor cells to treatment is closely related to the interaction of TME (100). In TME of ESCC, tumor-associated fibroblasts CAF expressing fibroblast activating protein (FAP) are distributed around the tumor. CAF attracts inhibitory TILs through paracrine signals and influences T cell penetration into the tumor center through direct physical interactions (101, 102). Within the TME, secretion of suppressive cytokines by the tumor leads to a progressive reduction in cytotoxic CD8^+^ T cells and NK cells, while concurrently promoting the expansion of Tex, Tregs, and regulatory B cells. Also, DCs exhibit maturation and functional defects. In contrast, patient samples that achieved pCR/MPR showed substantial immune activation and enrichment, which was able to reverse this TME. From this TME, we can observe the reactivation of Tex, self-activation of NK cells, positive feedforward loops of M1 macrophages with CD8^+^ T cells, and B-cell and T-cell interactions in the TLS. However, such reversal is rarely seen in samples of patients who achieved pPR/pNR (**Figure 6**). Therefore, some studies have classified TME into metabolic subtypes, mesenchymal, immune activated, and epithelial subtypes by characterized genes and noted that immune activated were associated with better prognosis and better pathological response (87).

To more finely delineate the landscape of TME in ESCC following nCIT, Yang et al. employed scRNA-seq and scTCR-seq to analyze samples from 18 patients with ESCC who underwent nCIT. The study divided the samples into four groups for analysis: pre-treatment, post-treatment, responders, and non-responders. Comparing post-treatment to pre-treatment, the study indicated minimal changes in the relative proportions of T cells, B cells, myeloid cells, and mast cells across all cellular constituents. However, significant changes were observed in epithelial cells, fibroblasts, endothelial cells, smooth muscle cells, and neurons. Among these, fibroblasts, endothelial cells, neurons, and smooth muscle cells increased significantly, while epithelial cells decreased. Similarly, when comparing responders to non-responders, the same effects were observed. This validated the overarching viewpoint of the paper. Furthermore, the study pointed out that in samples with responses and post-treatment, cell subpopulations CD8^+^ Tex-CXCL13, CD8^+^ Tex-STMN1, CD4^+^ Treg-TNFRSF4, tCAF-MMP11, Macro-SPP1, Macro-EREG, and Macro-STMN1 were significantly reduced, while CD8^+^ Tn-IL7R, Bmem_TNFSF13B cells, TLS, iCAF-CFD, iCAF-CXCL12, and iCAF-PLA2G2A were significantly increased in the parent cell populations (103).

On the other hand, spatial distance is also the basis for ensuring immune interactions within the tumor. Before nCIT, PD-1-positive cells were closer to tumor cells than PD-1- negative cells. CD4^+^ and CD8^+^ T cells that were 100 μm away from the tumor center were more likely to be activated with better immune response as well as having better correlation. Moreover, most DC and macrophage were distributed within 30 μm from the tumor cells, and patients with closer spatial proximity tended to have better immune responses (45). Wang et al. classified the post-treatment primary tumor into four patterns: Type I, Type II, Type III, and Type IV. In MPR or pCR cases, residual tumor cells were mainly found in the mucosa and submucosa, and less frequently in the lamina propria and epithelium, presenting a Type I pattern. In contrast, in pPR and pNR cases, most residual tumor cells were distributed in four layers, presenting Type IV (32).

## 5 Biomarker

The progression of cancer is characterized by heterogeneity, and the prospects for antitumor immunity within TME may vary among individual patients (31). Typical biomarkers, such as PD-L1 expression, microsatellite instability, and tumor mutation burden, seem to be inconsistent in predicting pathological responses in neoadjuvant therapies (37). We discuss recent studies on the alteration of TME in ESCC after nCIT and have compiled a list of the most recent relevant biomarkers. See **Table 2**.

**Table 2:** Biomarker.

Biomarker	Prognostic Value		Biomarker Source
	Pathological Response	Clinical Prognosis	
Muscularis Propria Invasion pCR/MPR ypN0	N/A	DFS +	Physiology
Ratio of CD8^+^ to FoxP3^+^ TILs	+	N/A	TME
CCR4/CCR6 Chemokine Model	–	OS -	TME
CD8^+^Tex-SPRY1	+	N/A	TME
Ratio of Foxp3^+^T cells to CD4^+^T cells	–	N/A	TME
TREM2^+^ TAMs	–	OS PFS -	TME
ctDNA Status at Baseline	+	N/A	Transcriptome
PLEK2, IFI6	–	N/A	Transcriptome
Macrophage Migration Inhibitory Factor	N/A	OS DFS -	Hematological Test
COCL19A1	+	N/A	Transcriptome
Spatial Distribution Patterns of CD4^+^PD-1^+^/CD8^+^PD-1^+^ cell	N/A	OS -	TME
LINC 02096	–	N/A	Transcriptome
Spatial Distribution Patterns of APCs	N/A	OS PFS +	TME
Intratumoural B cell Features	+	OS PFS +	TME

+, positive correlation; -, negative correlation; N/A not studied or no correlation; TME, tumor microenvironment; pCR/MPR, pathologic complete remission/pathologic major remission; OS, overall survival; PFS, progression-free survival; DFS, disease-free survival; LINC, Long Intergenic Non-coding RNA; APCs, antigen-presenting cells.

Nowadays, many articles have identified certain genes or constructed models to predict pathological responses or clinical prognoses from existing databases. Jia et al. identified that eRNAAC005515.1, an enhancer eRNA, is associated with the local immune environment of ESCC and maybe a new biomarker for ESCC prognosis (104). Ma et al. analyzed patients’ clinical information and proposed AST, d -d-dimer, and CEA as independent predictors of objective remission rate after nCIT (105). There are also some researchers, who proposed different biomarkers from new fields. Huang et al. analyzed baseline respiratory samples from patients and proposed to predict the pathological response of ESCA patients by detecting volatile organic compounds (VOCs) in exhaled gas (106). Xu et al. analyzed stool samples from patients for 16S ribosomal ribonucleic acid (rRNA) V3-V4 sequencing and proposed the classification characteristics of the gut microbiome as a potential biomarker for predicting pathological responses and adverse reactions (107). Given the limitations of existing biomarkers, these emerging and novel biomarkers undoubtedly fill an exciting gap in this area.

## 6 Discussion and future prospects

Here, we discuss the alterations in TME following neoadjuvant therapy combined with immunotherapy and potential biomarkers (108). Nowadays, advances in sequencing technology provide more details about immune infiltrating cell interactions and spatial distribution. Thus, we have summarized and discussed the major alterations and interactions of immune cells in the TME of ESCC. We also compiled predictive biomarkers for the prognosis of ESCC based on these alterations. These will better and more finely stratify patients, providing a clear direction for effective anti-tumor therapy.
